# Supplementary material for: A R2R3-MYB Transcription Factor, VvMYBC2L2, Functions as a Transcriptional Repressor of Anthocyanin Biosynthesis in Grapevine (Vitis vinifera L.)
Source: Molecules. 2018 Dec 27;24(1):92. doi: 10.3390/molecules24010092 (PMC6337365; doi:10.3390/molecules24010092)
Supplement: Supplementary file 1 [file molecules-24-00092-s001.pdf]

Supplementary data

Table S1 Sequence of the primers in the experiment for qPCR.

Gene name	Forward Primer 5' → 3'	Reverse Primer 5' → 3'
VvPAL1	aagaggttgagagcgcaagaga	actgagacaatccagaagaggg
VvDFR1	gaagctgacagatttggggttt	aggtttctcatgtgaaggggga
VvLAR1	tgtgggaaaagaagattgggag	tttatgaaaatgtcgtgcgtga
VvLDOX1	aaaggaagttggtgggatggaa	tgtggaggatgaaggtgagagc
VvUFGT1	gatatggcagcagagatgggg	tgcgtgagaagagcgagttta
VvMYBC2L2	tgagaagaaaagcacaaagaaaaga	gactgaggaagtgagctccagcaac
VvActin	cctcaaccccaaggccaacaga	accatcaccagaatccagcaca
NtCHI	gtcaggccattgaaaagctc	ctaatcgtcaatgccccaac
NtCHS	ttgttcgagcttgtctctgc	agcccaggaacatctttgag
NtFLS	gaacttgaagggaaaagggg	tccctgtaggagggaggatt
NtDFR	aaccaacagtcaggggaatg	ttggacatcgacagttccag
NtLAR	tcaaggtcctttacgccatc	acgaacctgcttctctttgg
NtANR1	catttgactttcccaaacgc	attgggcttttgagttgtgc
NtANR2	tgttcccacttgggatgata	tgcacctatactctgttagtggc
NtANS	tggcgttgaagctcatactg	ggaattaggcacacactttgc
NtUFGT	gagtgcattggatgcctttt	ccagctccattaggtccttg
NtAn1a	accattctcgaacaccgaag	tgctagggcacaatgtgaag
NtAn1b	cttgaacacttctcaaaccga	tgctagggcacaatgtgaag
NtActin	aatggaactggaatggtcaaggc	tgccagatcttctccatgtcatccca
